# Compassionate reappraisal and rumination impact forgiveness, emotion, sleep, and prosocial accountability

**DOI:** 10.3389/fpsyg.2022.992768

**Published:** 2022-11-17

**Authors:** Charlotte V. O. Witvliet, Sabrina L. Blank, Andrew J. Gall

**Affiliations:** Department of Psychology, Hope College, Holland, MI, United States

**Keywords:** accountability, compassionate reappraisal, empathy, forgiveness, flourishing, sleep, rumination

## Abstract

Sufficient sleep quality and quantity are important for biopsychosocial well-being. Correlational research has linked trait forgiveness to better sleep. Prior experimental evidence also demonstrated contrasting effects of offense rumination versus compassionate reappraisal on forgiveness and psychophysiological responses, suggesting the value of testing effects on sleep. The present study assessed 180 participants (90 M, 90 F). First, we replicated an individual difference model of forgiveness, rumination, depressed and anxious affect, and sleep. Second, we conducted a quasi-experiment inducing offense rumination and compassionate reappraisal on two consecutive nights. Compassionate reappraisal (vs. rumination) replicated past research by prompting more empathic, forgiving, positive, and social responses, with less negative emotion including anger. New findings revealed that compassionate reappraisal (vs. rumination) was also associated with faster sleep onset, fewer sleep disturbances, and fewer sleep impairing offense intrusions. The morning after compassionate reappraisal, participants reported less rumination and intrusive impact of the offense, with more hedonic well-being and accountability to others. Compared to rumination, compassionate reappraisal was associated with more empathy and forgiveness, better sleep, well-being, and prosociality.

## Introduction

Empirical research has demonstrated associations between forgiveness and psychosocial variables (see Fehr et al., 2010 meta-analysis). Further, experiments testing forgiving processes and inducing more forgiving states also induced calmer and less negative emotion while subduing associated physiological reactivity (see Witvliet et al., 2020 for a review). Large-scale representative research *via* survey in the US has associated forgiveness with better sleep (Toussaint et al., 2019). Sleep comprises a quarter to a third of people’s lives, is essential for optimal physical health (Grandner, 2017), mental health (Matsumoto et al., 2011; Buysse, 2014), emotional processing (Tempesta et al., 2018), self-regulation (Barber et al., 2013), and emotional regulation (Palmer and Alfano, 2017; Vandekerckhove and Wang, 2018). Overall, sleep influences comprehensive human flourishing that includes positive social connection (Léger et al., 2001). In the current research, we first assessed the relationship of trait forgiveness and sleep *via* rumination and negative affect (Stoia-Caraballo et al., 2008); we also tested self-regulation (Carey et al., 2004) and flourishing (Keyes, 2002). We then assessed whether inducing two different cognitive responses to an unresolved real-life offense—rumination in contrast to a compassionate reappraisal of one’s offender—would yield predicted reliable differences with comparatively higher state levels of empathy and forgiveness as well as positive and calmer emotion (Witvliet, 2020), prosocial accountability to others for one’s actions (Witvliet et al., 2022), hedonic and eudaimonic flourishing (Keyes, 2002), and sleep (Knutson et al., 2017) after compassionate reappraisal. We offer this work as an initial empirical investigation of a compassion-oriented cognitive approach delivered online that may give people an alternative to reliving an offense and its negative impact which still allows for holding one’s offender accountable (Witvliet, 2020).

### Rumination

Rumination that repetitively reviews one’s problems and emotions has been associated with depressive symptoms, anxious symptoms, and poor problem-solving (Nolen-Hoeksema et al., 2008; Aldao et al., 2010). Rumination has also played an important role in the empirical literature on sleep. Specifically, people high (vs. low) on trait rumination experienced more pre-sleep intrusive thoughts and poorer sleep quality (Guastella and Moulds, 2007). Additionally, elevated states of rumination (e.g., stress-induced rumination) have increased sleep onset latency (Zoccola et al., 2009). Rumination has had a significant association with reduced sleep quality even when controlling for depression and anxiety (Thomsen et al., 2003). To better understand the role of rumination in the relationship between trait forgiveness and sleep over the past month, Stoia-Caraballo et al. (2008) tested and found that anger rumination as well as depressed and anxious affect mediated the forgiveness-sleep association; we aimed to replicate their indirect effect model results. Further, we assessed correlations with self-regulation and with flourishing based on theorizing that rumination would have inverse associations, whereas forgiveness would have positive relationships with these variables (Witvliet, 2020).

Substantial experimental evidence has shown that rumination focused on a hurtful real-life offense and its negative impact (compared to cognitive coping responses consistent with holding the offender accountable) was associated with comparatively lower levels of self-reported empathy and forgiveness, as well as higher negative and aroused emotions and indicators of physiological stress (see Witvliet, 2020 for a summary; Witvliet et al., 2001, 2010, 2011, 2015). The current study aimed to assess self-reported sleep using a similar paradigm. Accordingly, we aimed to replicate experimental findings for rumination about a real-life offense versus a coping condition involving compassion to test differences between these two conditions on measures related to forgiveness, emotion, and social responses, while extending this to sleep. We offer this as a first experimental research step with a degree of ecological validity for understanding the human experience of ruminating about and coping with an interpersonal hurt before bed and associated results for forgiveness, psychosocial variables, and sleep.

### Reappraisal

Reappraisal is a cognitive approach to interpreting an emotional experience in a way that constructively addresses it (Watkins, 2008) while modulating one’s emotions through decreasing negative (Gross, 1998) and increasing positive emotions (Gross, 2007). Accordingly, reappraisal has been considered to be a regulation strategy that has been effective in emotional repair (Augustine and Hemenover, 2008); reappraisal has been associated with less depression and anxiety (Aldao et al., 2010), as well as more positive emotion, better relationships, satisfaction with life, and well-being (Gross and John, 2003).

Reappraisal has been examined in relation to rumination within the forgiveness literature (Witvliet, 2020). The most studied reappraisal approach is compassionate reappraisal of a real-life offender which responds to one’s offender in a way that aligns with theorizing about a process of forgiving an offender, which can involve:

1. Recognizing and responding to the humanity of the person responsible for the hurtful interpersonal injustice—not totalizing the wrongdoer in terms of the offense,
2. Acknowledging the wrongdoing and its impact—without minimizing,
3. Seeing the injustice as evidence that the wrongdoer needs positive change—responsibly correcting and improving behavior with respect to the relationship, and
4. Genuinely desiring the good of the person responsible for the wrongdoing—even when the relationship cannot be restored (Witvliet, 2020, p. 168).

In research testing compassionate reappraisal inductions, the person who has been hurt by another person’s offense has been prompted to focus on the humanity of the offender, to view the offense as an indication of that person’s need for learning, growth, or positive transformation, and to find a way to genuinely wish the offender well for their good–even if the relationship cannot continue (e.g., because it is unsafe, unwise, or not possible due to distance, differences, or death). Collectively, five experiments (Witvliet et al., 2010, 2011, 2015, 2020; Baker et al., 2017) have shown that compared to a condition prompting rumination about the offense and its impact, the condition of compassionate reappraisal has prompted higher state levels of self-reported empathy and forgiveness on scales and ratings, along with higher ratings of positive valence and perceived control, as well as lower levels of arousal and anger. Compassionate reappraisal has also been found to prompt more language use consistent with forgiveness and positive emotion, as well as sociality. Accordingly, we aimed to replicate and extend the experimental findings for compassion in comparison to rumination. Specifically, in alignment with Stoia-Caraballo et al.’s (2008) findings, we tested effects on sleep and anticipated better sleep after compassionate reappraisal vs. rumination. Additionally, self-compassion has been shown to improve sleep quality by reducing self-blame and the utilization of cognitive emotional regulation strategies (Semenchuk et al., 2021). Here, rather than examining self-compassion, we focused on other-oriented compassionate reappraisal of an offender.

We also examined the effects of compassionate reappraisal (vs. rumination) on prosociality and flourishing (hedonic and eudaimonic) the next morning. Recent research found that accountability and flourishing were both associated with forgiveness at the trait level (Witvliet et al., 2022). Compassionate reappraisal can align with holding offenders accountable for wrongdoing (Witvliet, 2020); yet, no prior research has tested whether compassionate reappraisal (vs. rumination) is associated with a greater willingness to be accountable to others. We reasoned that because experiment participants have been more forgiving, empathic, and social—as well as more positive—after compassionate reappraisal (vs. rumination; Witvliet et al., 2010, 2020; Baker et al., 2017), they would also be likely to be more prosocial in welcoming their accountability to treat others responsibly. Further, substantial evidence has shown that after compassionate reappraisal (vs. rumination), participants experienced a shift from negative and aroused emotion to more positive and calm emotion with more social language (Witvliet et al., 2010, 2015, 2020). This set of positive hedonic emotions and sociality led us to assess whether greater state flourishing would also occur on a measure of feeling and functioning well (Keyes, 2002).

### Current study

The purpose of this study was to advance the science and practice of positive psychology with respect to forgiveness and sleep. We first evaluated forgiveness and sleep related variables at the individual difference (trait) level to replicate past findings (Stoia-Caraballo et al., 2008) and incorporate self-regulation and flourishing measures (Witvliet, 2020). Then we extended the quasi-experimental literature that has tested in the moment (state) forgiveness levels to assess whether adopting a compassionate reappraisal approach—which has been associated with forgiveness and psychophysiological side effects (compared to rumination; Witvliet et al., 2010, 2011, 2015; Baker et al., 2017)—would also benefit sleep. We assessed measurements across two consecutive night-morning pairs that spanned a total of three consecutive weekdays. First, we assessed individual difference level variables. Second, we used a quasi-experimental design to test the effects of induced rumination and compassionate reappraisal about a real-life unresolved interpersonal offense on forgiveness and that night’s sleep. Drawing on the literature, participants began with rumination on night one, and then engaged compassionate reappraisal on night two. We followed this sequence because (1) interventions such as REACH (see Worthington, 2020) begin with recalling and reliving a real-life interpersonal offense before engaging empathy toward the offender, (2) compassionate reappraisal is known to evoke empathic change on the first trial and to transform subsequent rumination with greater empathy toward one’s offender (Witvliet et al., 2015), and (3) ethically, we had to conclude the study with compassionate reappraisal rather than rumination because rumination has known adverse effects of on negative affect and stress physiology, whereas compassionate reappraisal has known psychophysiological benefits (Witvliet, 2020).

We designed this research to be conducted using remote online technology (in keeping with the methodology of this special section for compassion research using online methods) to deliver conditions instructions adapted from the paradigm used by Witvliet et al. (2020). This approach made the intervention study accessible to participants in their natural sleep environment using well studied measures. This research did not use physiological assessments of sleep variables (e.g., actiwatches, ambulatory monitors) due to cost and challenges in the pandemic; thus, we invite other researchers to incorporate quality objective sleep-related measures in follow-up research.

We tested an equal number of self-identified male and female participants given gender variations in sleep quality and quantity (Jonasdottir et al., 2021). We also focused on college students because they experience a confluence of developmental and life factors that contribute to impaired sleep quality and quantity, through difficulty falling asleep, social pressures, and elevated risk of anxiety and depression (Garcia, 2021).

#### Hypotheses

At the individual difference (trait) level, we preregistered the following primary hypotheses.^1^ Better baseline sleep quality and sleep quantity would have a significant inverse relationship with trait rumination and negative affect in accordance with research by Mellman (2006), but a direct relationship with trait self-regulation, forgiveness, and flourishing in the past month. The latter hypotheses extend from research on sleep and its beneficial associations with self-regulation (Barber et al., 2013), emotion regulation (Palmer and Alfano, 2017; Vandekerckhove and Wang, 2018), emotional processing (Tempesta et al., 2018), forgiveness (Toussaint et al., 2019), and quality of life (Léger et al., 2001).

We also hypothesized that forgiveness would have a significant inverse relationship with trait rumination and negative affect, but a direct relationship with trait self-regulation and flourishing. This is consistent with theorizing by Witvliet (2020) based on experimental data showing that rumination (vs. compassionate reappraisal) was associated with lower self-reported forgiveness, greater negative affect, and cardiac dysregulation, whereas coping conditions that induced forgiveness also reduced negative affect and have maintained cardiac regulation similar to baseline levels (Witvliet et al., 2011, 2015); further, recent research has found a correlation between forgiveness and flourishing (Witvliet et al., 2022). Stoia-Caraballo et al. (2008) found that forgiveness indirectly predicted sleep quality through rumination and negative affect (and through negative affect alone); thus, we predicted that we would replicate the model results.

At the state level, we made predictions based on programmatic research on compassionate reappraisal and rumination, including measures used by Baker et al. (2017) and Witvliet et al. (2010, 2011, 2015, 2020). Accordingly, we predicted that compassionate reappraisal (vs. rumination) toward a real-life offender would prompt higher empathy and forgiveness scores (Witvliet et al., 2010, 2015, 2020; Baker et al., 2017). Consistent with these studies, we also hypothesized that compared to compassionate reappraisal, the offense rumination condition would prompt more negative and aroused emotion—including anger as well as less perceived control (Witvliet et al., 2010, 2011), and lower scores for that night’s sleep quality and quantity (Guastella and Moulds, 2007). Compared to compassionate reappraisal, we also hypothesized that rumination would prompt higher scores on the intrusive impact of events and rumination scales because repetitive thoughts may continue including during sleep (Horowitz et al., 1979; McCullough et al., 1998), as well as lower self-regulation, accountability to others, and flourishing scores the subsequent morning. The self-regulation hypothesis is based on research by Witvliet et al. (2010, 2011) noting that rumination (vs. compassionate reappraisal) was associated with significantly lower heart rate variability (vs. no difference) compared to baseline levels. The state accountability and flourishing hypotheses were based on theorizing (Witvliet, 2020) and data (Witvliet et al., 2022) linking the relational virtues of forgiveness and accountability with each other and with flourishing as feeling good and functioning well in relationships with a sense of purpose (Keyes, 2002).

## Materials and methods

### Participants

One-hundred and eighty undergraduate students from a midwestern United States undergraduate liberal arts college participated in the current study as one way to receive extra credit or meet course expectations for research experience. As documented in the OSF pre-registration, we aimed to obtain a gender-balanced sample as close to 200 as possible based on a G*Power analysis considering the possibility of small effect sizes for sleep and the possibility that participant drop-out could occur for this 4-part repeated measures study. The gender distribution of these 180 participants was 90 self-identified females and 90 self-identified males. Upon obtaining a sufficient sample of self-identified females, we recruited participation of self-identified males to aim to have a gender balanced sample. Of these 180 participants, 153 participants (85.0%) self-identified as *White, Anglo, Caucasian, or European American*; five (2.8%) as *Hispanic, Latino, or Spanish origin*; eight (4.4%) as Black or African American, 13 (7.2%) as *Asian or Asian American*; and one (0.1%) identified *Some other race or ethnicity or origin*. The 180 participants completed the trait measures on night one, and 170 participants individually identified and wrote about a real-life offender for the experimental conditions on night one and night two, and also completed all components of the experiment on night one, morning one, night two, and morning two.

To handle missing data, we adopted the following strategies. If a scale item was unanswered, we used mean imputation based on that participant’s other responses on that scale (only 4 items were missing across all participants, thus we imputed 0.008% of scale items). If a single-item rating was missing, it was excluded from analysis (only 9 items were missing across all participants, thus we excluded 0.171% of single-item ratings). One participant completed all components of the experiment, except the anxiety scale component of the depression anxiety stress scale (DASS anxiety) on night one; for this participant, we excluded the DASS anxiety score, but we included all other data in analyses.

### Overall design

We used a correlational survey design to assess individual differences, followed by an experiment with an incomplete repeated-measures within-subjects design to test the effects of rumination vs. compassionate reappraisal toward a specific offender on two consecutive evening-morning periods. Each participant selected a particular previous, non-traumatic and unresolved interpersonal offense where they felt hurt or wronged, focusing on this single offense for both experiment conditions. Readers are directed to the protocol materials to see the imagery and writing prompts for each condition (see pre-registration^1^ and its associated project link ^3^ for all protocol and measures materials as well as de-identified data).

On the first night, participants completed the rumination induction beginning with a two-minute imagery period in which they actively focused on the negative thoughts, feelings, and physical responses they experienced as they thought about the ways they experienced harm by the offender. After this, they responded to written response prompts about their emotions, blame of the offender, harm experienced, and continued impact of the offense (Witvliet et al., 2020).

On the second night, participants completed the compassionate reappraisal condition beginning with a two-minute imagery period, focusing on the offender’s humanity and need for positive transformation, trying to genuinely wish them well with compassion. Participants then responded to writing prompts about the offender’s humanity, the wrongdoing as evidence of the offender’s need for positive change and growth, a small way to wish the person well, and how one’s compassion can be genuine even if the relationship discontinues.

All participants were in the rumination condition on night one and compassionate reappraisal condition on night two; this sequence aligned with the intervention literature (Worthington, 2020), as well as evidence that rumination ought to be assessed first because compassionate reappraisal elevates empathy for subsequent rumination (Witvliet et al., 2015), and ethical considerations to avoid the potential harm of ending a study with rumination which has been associated with negative affect and physiological stress (Witvliet, 2020).

### Procedure

Participants were recruited for this Human Subjects Review Board-approved research through online software (Sona Systems).^4^ They then completed all phases of this study online, with informed consent, data collection, and debriefing conducted using Qualtrics software (Provo, UT). Participants completed the study either Monday night through Wednesday morning, Tuesday night through Thursday morning, or Wednesday night through Friday morning. Data were not collected on weekends due to the potential confound of different sleep—wake schedules on weekends within this US sample of college students (Machado et al., 1998).

The study began at 8:00 pm on night one on Monday, Tuesday, or Wednesday night to ensure sufficient time to complete the surveys before participants went to bed. Both nights, participants received an email with the Qualtrics link at 7:45 pm to complete the study between 8:00 pm and 11:59 pm. We note that the preregistered plan indicated that we would allow completion between 8:00 pm and 9:00 pm, however, the longer timeframe was more feasible for participants’ evening schedules.

Once participants clicked on the night one link, they completed informed consent, as well as demographics and individual difference measures of sleep quality and quantity, flourishing, forgiveness, negative affect, rumination, and self-regulation. Next, participants identified a specific person they held responsible for an unresolved interpersonal offense against them. They then underwent the rumination manipulation. This section was timed in Qualtrics, so participants could not proceed until they completed the rumination condition for the full 2 min. Following rumination imagery, participants were prompted to write about their thoughts, feelings, and reactions to the event through a variety of free response questions, and then they were required to sign a safeguard statement which provided mental health resources to ensure protection. Finally, after the rumination imagery and writing on night one, participants completed scales and ratings of their state levels of emotions, empathy, and forgiveness.

The next day, the Qualtrics questionnaire link for morning one was emailed to participants at 6:00 am, which they were instructed to complete upon awakening before noon. Participants then completed surveys that evaluated their sleep disturbances the prior night, levels of the perceived impact of the offense event, and levels of rumination about it since the imagery and writing they did the night before. Levels of state self-regulation, willingness to be accountable to others, and flourishing were also assessed.

On night two, after instructions were emailed at 7:45 pm, participants again had between 8:00 pm and 11:59 pm to complete this portion of the study. During this period of time, the compassionate reappraisal manipulation was completed. As before, they were required to consider the same offense as night one (i.e., rumination condition), but this time to utilize the provided compassionate reappraisal techniques for 2 min before proceeding. Participants were prompted to engage in compassionate imagery and to write down their thoughts, feelings, and reactions through free-response questions. They were prompted to agree to the same safeguard statement as provided in night one. Additionally, participants’ state levels of emotions, empathy, and forgiveness were assessed with scales and ratings.

The following (second) morning, participants received a 6:00 am email link prompting them to report by noon their prior night’s sleep disturbances, their perceived impact of the offense event and rumination about it since their imagery and writing the night before, as well as state self-regulation, accountability to others, and flourishing. Following completion of the study, participants were debriefed about the study and offered follow-up mental health resources available by phone and telehealth.

### Measures

All reported measures for this registered study are publicly available (see study registration^5^ and associated project link^6^ to select Protocol files). Below we report the measures we analyzed and provide the citations for them. Cronbach’s alphas are reported for the current sample. For state measures, the alpha for the rumination condition is reported before the alpha for the compassionate reappraisal condition.

### Trait measures (measured on night one before rumination)

#### Sleep quality and quantity (PSQI)

Baseline measures of sleep quality and quantity were evaluated utilizing the Pittsburgh Sleep Quality Index (PSQI), a questionnaire detailing the participant’s self-reported sleep patterns within the past month; the scale has items for a bed partner or roommate’s responses, but these do not affect the global score and were not used. The PSQI includes open-ended sleep hygiene questions, such as “*When have you usually gone to bed?”* as well as ordinal questions where participants rate the extent of disturbance by variables that influence sleep quality (e.g., *In the past month, how often have you had trouble sleeping because you feel too cold*) on a scale of 0 (*not during the past month)* to 3 (*three or more times per week*). The PSQI global score is a total of seven components: *subjective sleep quality, sleep latency, sleep duration, habitual sleep efficiency, sleep disturbances, use of sleeping medication, and daytime dysfunction*. The possible range of global scores for the PSQI is zero to 21, with a higher score indicating poorer sleep quality. The PSQI is considered the gold standard for measuring self-reported sleep quality and quantity (Buysse et al., 1989). In this sample, the internal consistency of the global PSQI score across all seven components (⍺ =0.67) was similar to recent research by Zhang et al. (2020).

#### Short Form Self-Regulation Questionnaire (SSRQ)

We assessed trait self-regulation using the 31-item questionnaire developed by Carey et al. (2004), evaluating the extent to which individuals felt competent in their ability to regulate their behaviors while pursuing goals (*α* = 0.84). Participants were asked to rate their level of agreement with several statements pertaining to self-regulatory behaviors, such as “*I do not seem to learn from my mistakes*” (reversed) and “*I have a lot of willpower*” on a scale of 1 (*strongly disagree*) to 5 (*strongly agree*). Scores can range from 31 to 155, with a higher score indicating higher levels of self-regulation.

#### Ruminative Response Scale (RRS)

We used the 22-item RRS by Nolen-Hoeksema et al. (1999), which details the frequency of ruminative thoughts including when feeling sad, down, or depressed (⍺ = 0.95). Statements include rating the frequency of thoughts such as “*Think about how alone you feel*” and “*Think about all your shortcomings, failings, faults, mistakes*” on a scale of 1 (*almost never*) to 4 (*almost always*). Scores can range from 22 to 88, with a higher score indicating higher levels of self-regulation.

#### Depression Anxiety Stress Scale (DASS)

Negative affect was evaluated using 14 depression items and 14 anxiety items on the DASS (Lovibond and Lovibond, 1995), a scale that assessed the presence of depressive and anxious symptoms over the past week (*α* = 0.96). Participants rated statements indicative of symptoms of depression, such as (in the past week) “*I felt that I had nothing to look forward to*,” and statements indicative of symptoms of anxiety, such as “*I felt scared without any good reason*” on a scale of 0 (*did not apply at all*) to 3 (*applied very much or most of the time*). Scores can range from 0 to 42, with a higher score indicating greater levels of depressive and anxious symptoms within the past week.

#### Trait Forgiveness Scale (TFS)

Dispositional forgiveness toward other people was assessed using the 10-item Trait Forgiveness Scale by Berry et al. (2005; *α* = 0.79). Participants were asked to rate the extent of agreement to statements such as “*I can forgive a friend for almost anything”* and “*I have always forgiven those who have hurt me*” on a scale of 1 (*strongly disagree*) to 5 (*strongly agree*). Scores can range from 10 to 50, with a higher score indicating higher level of dispositional trait forgiveness.

#### Flourishing Scale (FS)

Levels of flourishing over the past month were evaluated using Keyes’ (2002) 14-item scale which combines hedonic items about feeling good and eudaimonic indicators of functioning well (*α* = 0.94). Questions include rating the extent to which one has felt experiences consistent with hedonic flourishing such as “*happy*” and “*interested in life,*” and eudaimonic flourishing such as “*I have something important to contribute to society*” and “*My life has a sense of direction or meaning to it*” on a scale from 1 (*never*) to 6 (*every day*). Scores can range from 14 to 84, with a higher score indicating a greater experience of flourishing within the past month.

### State measures (measured on night one and night two during and after experimental conditions)

#### Night state measures

We report the analyzed measures in the order in which participants received them. Because the protocol provides all measures, we provide wording and sample items only where we believe it will offer needed clarity for readers.

#### Linguistic Inquiry and Word Count (LIWC)

After the imagery and writing prompts for each condition (rumination, compassionate reappraisal), participants were instructed to *Write a paragraph (60+ words). If the person who hurt or offended you walked into the room right now, what would you feel like saying or doing in response to him/her?* The word count suggestion was offered to guide participants on length for responses; however, a minimum word count was not required, and no participants wrote nonsensical responses or unrelated filler content. We used LIWC software (Pennebaker et al., 2007) to compute the proportion of words in participant responses that corresponded with software’s internal dictionary lists of positive emotions, negative emotions and social words, plus a forgiveness dictionary developed by Witvliet et al. (2010). This allowed us to test for differences in word use to describe responses to one’s offender immediately after inductions of rumination and compassionate reappraisal.

#### Spielberger’s State Anger Scale (SAS)

State levels of anger in participants were evaluated using Spielberger’s 10-item State Anger Scale (Spielberger, 1988; *α*s = 0.94, 0.95), which includes several statements related to anger such as “*I am mad*” and “*I feel like banging on the table*,” rated from 1 (*strongly disagree*) to 4 (*strongly agree*). Scores on the scale can range from 10 to 40, with a higher score indicating greater state levels of anger.

#### Ratings

Participants provided single item ratings based on Witvliet et al.’s (2001) approach, which has been adopted in subsequent studies comparing offense rumination and compassionate reappraisal conditions (Witvliet et al., 2010, 2011, 2015, 2020; Baker et al., 2017). They rated the valence of their emotion right after each condition, from (1) *Very negative* to (7) *Very positive*. They also rated how aroused/intense, in control, angry, and sad they felt, as well as how much empathy and forgiveness they felt for the person who hurt them, from (1) *Not at all* to (7) *Completely* (e.g., Witvliet et al., 2020).

#### Batson’s Empathy Adjectives Scale

We used Batson et al.’s (1986) 8-item empathy adjectives scale to assess state empathic emotions for the person who hurt them (*α*s = 0.91, 0.93). This scale has been established as a valid and reliable way to assess empathy levels, and has been utilized in other studies pertaining to empathy and forgiveness (e.g., Kidwell, 2009; Niezink et al., 2012; Witvliet et al., 2020). Participants indicated the extent they experienced affective states for their offender, such as “*sympathetic*,” “*moved*,” and “*compassionate*” on a scale of 0 (*not at all*) to 5 (*extremely*). Total scores can range from 0 to 40, with higher scores indicating greater levels of state empathy toward one’s offender.

#### Transgressions-Related Interpersonal Motivations Inventory (TRIM-18R)

State levels of forgiveness were assessed through the 18-item scale by McCullough et al. (2006). This questionnaire examines benevolent, avoidance, and revenge motivations by rating the extent to which one agrees with statements such as “*I’ll make him/her pay*” and “*I do not trust him/her*” on a scale of 1 (*strongly disagree*) to 5 (*strongly agree*). Total scores (for which avoidance and revenge items were reverse-scored) can range from 18 to 90; higher scores indicate greater overall state forgiveness toward one’s real-life interpersonal offender (*α*s = 0.89, 0.92).

#### Morning state measures

In order to assess the sleep quality and quantity of the prior night, as well as other psychological indicators, participants completed the following measures each morning upon awakening.

#### Sleep Health

We used a modified version of the Sleep and Health Self Report Scale from the National Sleep Foundation. The Sleep Health Self Report Scale includes three domains of sleep health: sleep quantity (using questions such as, *I went to bed last night at: ____, I woke up this morning at:_____*), sleep quality (using questions such as, *When I woke up for the day, I felt …* 1 (*very fatigued*) to 5 (*very refreshed*), and sleep disturbances (using questions such as, *Last night, my sleep was disturbed by: Noise, Lights, Pets, Allergies, Temperature, Discomfort, Stress, Anxiety, Anger, Sadness, Other, or None*). We only used two of these three domains (sleep quantity and sleep quality), because the sleep disturbances could be measured more accurately with the Sleep Disturbance Questionnaire (SDQ). The Sleep and Health Self Report Scale has been shown to be a valid measure and has been used by other researchers to assess sleep health (Knutson et al., 2017). Several studies have examined self-reported sleep quantity and sleep quality using items similar to the items in the current study assessing sleep on the night before (Leigh et al., 1988; Meltzer et al., 2012) and using a repeated-measures design (Ward et al., 2008).

#### Sleep Disturbance Questionnaire (SDQ)

We used Espie et al.’s (1989) 12-item questionnaire which assesses cognitive, emotional, physical, and behavioral disruptions to one’s sleep the night before (⍺s = 0.91, 0.91). Participants were prompted to rate the extent to which they experienced varying disturbances, such as “*I could not get into a comfortable position in bed*” and “*I got too worked up at not sleeping*” on a scale of 1 (*never true*) to 5 (*very often true*). Scores can range from 12 to 60, with a higher score indicating a greater amount of sleep disturbances from the prior night. This scale has been used to evaluate the impact of cognitive factors on patients with sleep disturbances and disorders such as insomnia (Espie et al., 2000).

#### Impact of Events Scale (IES)

We used the 7-item intrusion subscale of the IES (Horowitz et al., 1979) to assess how much participants were distressed by intrusive thoughts, images, and dreams since the offense-focused imagery and writing they did the prior evening (⍺s = 0.90, 0.89). Items included rating statements such as “*Other things kept making me think of it*,” “*I had dreams about it*” and “*I had trouble falling asleep or staying asleep because of pictures or thoughts about it that came into mind*” on a scale of 0 (*not at all*) to 4 (*extremely*). Scores can range from 0 to 28, with a higher score indicating a greater perceived impact of the event.

#### Rumination About an Interpersonal Offense (RIO)

We used Wade et al.’s (2008) 6-item scale to assess rumination about their offender and offense since the induction of imagery and writing the prior night (⍺s = 0.91, 0.92). Items include rating statements such as “*the wrong I suffered is never far from my mind*” and “*I cannot stop thinking about how I was wronged by this person*” on a scale of 1 (*strongly disagree*) to 5 (*strongly agree*). On a scale of 6 to 30, a higher total score indicates greater levels of rumination about the offense.

#### Self-Regulation Scale (SRQ)

We administered an unpublished 25-item questionnaire by Twenge and Ciarocco (2004) assessing the extent to which individuals felt able to manage their present emotions, thoughts, and actions (*α*s = 0.95, 0.96). Items include rating statements such as “*I feel drained*” and “*I would want to quit any difficult task I was given*” on a scale of 1 (*strongly disagree*) to 4 (*strongly agree*). A higher total score indicates greater levels of self-regulation, with a total possible range of scores of 25 to 100.

#### Accountability scale (state version)

We adapted an 11-item measure of the disposition to welcome one’s accountability to others (Witvliet et al., 2022) so we could assess participants’ current state levels of the prosocial virtue. Participants were instructed to assess their current responsibilities in relation to others (e.g., *Right now…I am willing to be held responsible for my contributions on tasks*, *I care about the people who are affected by my work*, *I welcome corrective feedback from people who evaluate me)* using a 1 (*strongly disagree*) to 5 (*strongly agree*) Likert-type scale (*α*s = 0.88, 0.90). Scores can range from 11 to 55, with higher scores indicating greater state levels of welcoming relational accountability.

#### Flourishing

State levels of flourishing as feeling good (hedonic well-being) and functioning well (eudaimonic well-being) were evaluated using a state adaptation of Keyes’ (2002) flourishing measure reported above, which has been used in psychiatric inpatients (Currier et al., 2019). Participants rated *Right now, I am… happy*, *interested in life*, and *satisfied* (hedonic items) as well as 11 eudaimonic items (e.g., *I feel that I have something important to contribute to society,* and *that I belong to a community*) on a 0 (*not at all*) to 4 (*completely*) response scale (*α*s = 0.92, 0.93). Scores can range from 0 to 56, with a higher score indicating greater overall state levels of flourishing.

### Data analysis

Data were analyzed using SPSS 27. In addition to bivariate Pearson correlations, we tested an indirect effect model using the PROCESS 3.5 Macro (Hayes, 2017) for SPSS 27, with two mediators in Model 6, using 5000 bootstraps and 95% confidence intervals. Specifically, we assessed the indirect effect of forgiveness on sleep quality through rumination and negative affect, using the PROCESS add-on for SPSS. For the quasi-experiment, we performed repeated measures analyzes of variance (ANOVAs) to compare the rumination condition to the compassionate reappraisal condition for all dependent variables. Finally, to assess the internal consistency of the scales utilized, we used scale reliability analysis in SPSS.

## Results

De-identified data for this registered study are publicly available (see study registration^8^ and associated project link^9^ to select Data files).

### Individual differences

**Figure 1:**
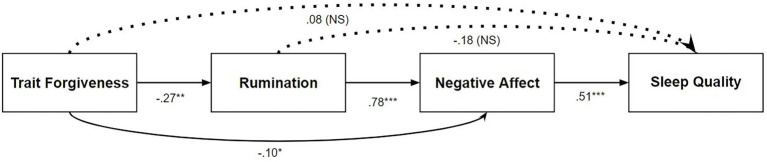
We tested an indirect effect of trait levels of forgiveness on sleep quality through trait rumination and negative affect in PROCESS 3.5.3 using Model 6. The measures used in the model, from left to right were the Trait Forgiveness Scale (TFS), the Ruminative Response Scale (RRS), the combined Depression Anxiety Stress Scale (DASS) assessing negative affect, and the Pittsburgh Sleep Quality Index (PSQI). We report standardized coefficient *betas* for direct paths tests, followed by the completely standardized effects for the two reliable indirect effects. Trait forgiveness was associated with trait rumination (*b* = −0.27, *SE* = 0.17, 95% *CI* = −0.94 to −0.28), rumination was related to negative affect (*b* = 0.78, *SE* = 0.05, 95% *CI* = 0.75–0.95), and negative affect was related to sleep quality (*b* = 0.51, *SE* = 0.004, 95% *CI* = 0.01–0.03). The path from trait forgiveness directly to negative affect was also significant (*b* = −0.10, *SE* = 0.11, 95% *CI* = −0.48 to −0.03). The path from trait rumination to sleep quality was not significant (*b* = −0.18, *SE* = 0.004, 95% *CI* = −0.02 to 0.002). Further, the direct path from trait forgiveness to sleep quality was not significant (*b* = 0.08, *SE* = 0.01, 95% *CI* = −0.01 to 0.02). Overall, as predicted, the model showed that trait forgiveness was associated with sleep quality *via* two significant indirect effects: through both trait rumination and negative affect (*b* = −0.11, *SE* = 0.04, 95% *CI* = −0.18 to −0.04), and through negative affect alone (*b* = −0.05, *SE* = 0.02, 95% *CI* = −0.09 to −0.01).

Table 1 shows the correlations results. Consistent with predictions, lower PSQI global scores (indicating better sleep quality) were associated with lower levels of rumination, as well as the DASS negative affect measures of anxiety and depression, and higher self-regulation and flourishing. Forgiveness also had a significant inverse correlation with trait rumination, anxiety, and depression, but a direct positive correlation with trait self-regulation and flourishing. PSQI scores did not show the predicted significant correlation with trait forgiveness; rather, the association of forgiveness and sleep was indirect. Specifically, this study replicated Stoia-Caraballo et al.’s (2008) indirect effect of trait forgiveness on sleep quality as described and depicted in Figure 1. That is, trait forgiveness predicted better sleep on the PSQI through lower rumination and negative affect (and through lower negative affect only).

**Table 1:** Means, standard deviations, and correlations for sleep quality and quantity, and trait measures.

Variable	*M*	*SD*	1	2	3	4	5	6
1. PSQI	6.61	3.11						
2. SSRQ	113.51	16.94	−0.256^**^					
3. RRS	47.28	15.70	0.411^***^	−0.449^***^				
4. Anxiety	9.01	8.18	0.399^***^	−0.304^***^	0.662^***^			
5. Depression	11.04	10.43	0.453^***^	−0.530^***^	0.782^***^	0.669^***^		
6. Forgiveness	36.00	6.80	−0.137	0.290^***^	−0.257^**^	−0.259^***^	−0.302^***^	
7. Flourishing	57.18	14.55	−0.349^***^	0.589^***^	−0.584^***^	−0.482^***^	−0.746^***^	0.316^***^

M and SD represent mean and standard deviation, respectively. PSQI, Pittsburgh Sleep Quality Index (lower scores indicate better sleep); SSRQ, Short Form Self-Regulation Questionnaire; RRS, Ruminative Response Scale. Anxiety and Depression are from the Depression and Anxiety Stress Scale (DASS). Possible scale score ranges are reported in the Measures section of the Method.

**Indicates *p* ≤ 0.01.

*** Indicates *p* < 0.001.

### Induced rumination versus compassionate reappraisal

All reported results and interpretations are focused on the dependent variables for these two conditions in comparison to each other. We do not make claims about how these variables would compare to nights in which participants were not thinking about their unresolved offense and real-life offender or any other context for sleep.

#### Written responses about the offender

Table 2 reports the repeated-measures ANOVA results for linguistic analyzes of participants’ written descriptions of how they would respond to encountering their offender right after each condition. As predicted, LIWC data showed that after compassionate reappraisal (vs. rumination), participants used a significantly higher proportion of words associated with forgiveness, positive emotions, and sociality, whereas rumination prompted use of significantly more negative emotion words.

**Table 2:** Night measurement means, standard deviations, *F* values, 0.95 confidence intervals for condition mean differences, significance, and partial eta-squared effect sizes.

Variable	Rumination *M* (*SD*)	Compassionate reappraisal *M* (*SD*)	*F*	Mean difference 0.95 *CI*	*p*	*η^2^*
Linguistic inquiry and word count (LIWC)						
Forgiveness	0.22 (0.51)	0.44 (0.83)	8.62	−0.36, −0.07	0.004	0.049
Positive emotion	3.31 (2.18)	4.61 (3.33)	21.30	−1.85, −0.74	<0.001	0.112
Negative emotion	2.99 (2.15)	2.26 (1.97)	11.30	0.30, 1.16	0.001	0.063
Social	14.37 (4.31)	15.46 (4.94)	5.85	−1.99, −0.20	0.017	0.033
State self-report scales and ratings						
Anger scale	18.39 (7.26)	13.55 (5.22)	102.12	3.89, 5.78	<0.001	0.377
Negative-to-positive rating	3.56 (1.43)	4.74 (1.30)	112.74	−1.40, −0.96	<0.001	0.400
Arousal rating	3.29 (1.67)	2.19 (1.31)	77.03	0.85, 1.35	<0.001	0.313
Perceived control rating	4.87 (1.74)	5.26 (1.67)	8.55	−0.65, −0.13	0.004	0.048
Sadness rating	3.92 (2.00)	2.95 (1.66)	51.44	0.70, 1.23	<0.001	0.233
Empathy scale	10.96 (8.66)	16.35 (10.03)	122.98	−6.35, −4.43	<0.001	0.421
Forgiveness scale	59.83 (13.05)	63.77 (13.03)	46.25	−5.09, −2.80	<0.001	0.215

Single item ratings for anger, empathy, and forgiveness showed the same reliable differences between rumination and compassionate reappraisal as each of their respective scales (all *F*s ≥ 52.62, all *p*s ﹤ .001, and all *𝜂*^2^s ≥ 0.24). Possible score ranges are reported in the Measures section of the Method.

#### Self-reported forgiveness and emotions

Additionally, Table 2 documents the scales and ratings results, which were consistent with hypothesized patterns. Following the rumination (vs. compassionate reappraisal) condition, participants experienced more state anger, arousal, and sadness. By contrast, compassionate reappraisal activated more empathy, forgiveness, perceived control, and more positively valent emotion.

#### Sleep effects that night

**Figure 2:**
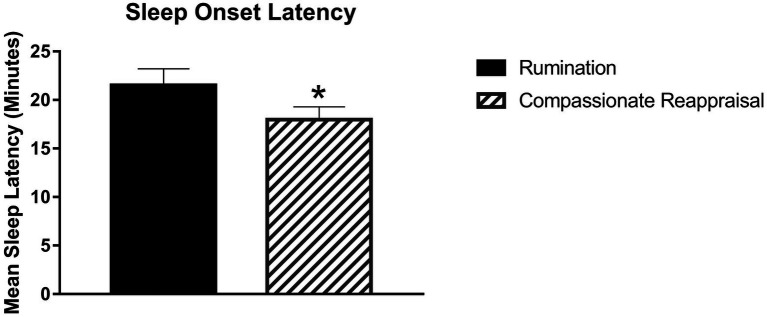
Sleep onset latency was significantly shorter indicating participants fell asleep faster following the compassionate reappraisal manipulation (*M* = 17.96, *SEM* = 1.35) compared to after the rumination manipulation (*M* = 21.51, *SEM* = 1.70), *F* (1, 169) = 4.15, *p* = 0.043. Error bars indicate Standard Error of the Mean (SEM). ^*^Indicates *p* < 0.05.

**Figure 3:**
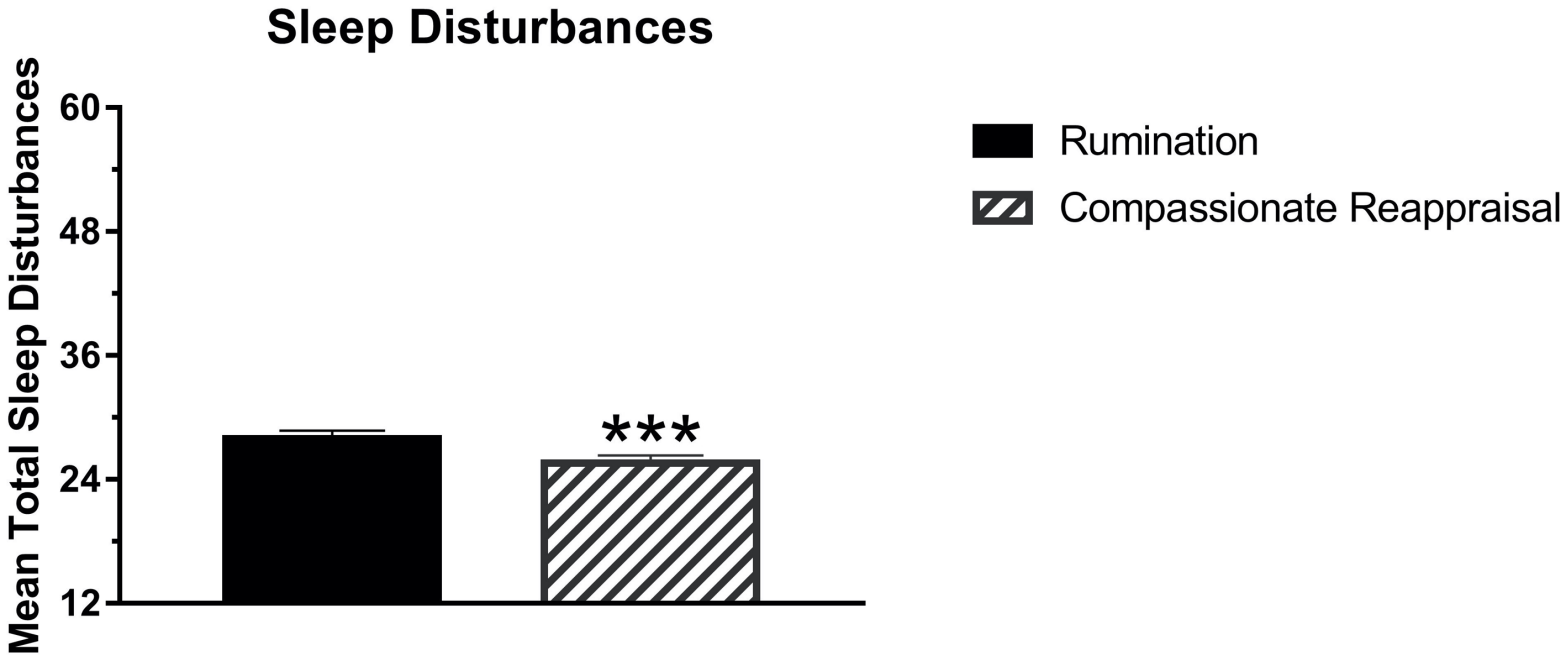
Total sleep disturbances as measured by the Sleep Disturbances Questionnaire (SDQ) were significantly lower following compassionate reappraisal (*M* = 25.57, *SEM* = 0.77) compared to rumination (*M* = 27.95, *SEM* = 0.81), *F* (1, 169) = 11.70, *p*   < 0.001. Error bars indicate Standard Error of the Mean (SEM). The y-axis gives the minimum to maximum score on the SDQ. ^***^Indicates *p* < 0.001.

Several findings were consistent with the hypothesis that evening rumination (vs. compassionate reappraisal) would be followed by lower sleep quality and quantity for that night’s sleep. As Figure 2 demonstrates, sleep onset latency was delayed following the rumination (vs. compassionate reappraisal) condition; that is, participants fell asleep faster following the compassionate reappraisal condition. Additionally, Figure 3 shows that participants reported reliably more sleep disturbances on the SDQ following the rumination condition (vs. compassionate reappraisal). Table 3 further documents that after ruminating about their offense experience, participants reported more *trouble falling or staying asleep because of pictures or thoughts about it that came into mind* (IES item 2). Although no analyses directly countered predictions, the following sleep variables did not show reliable differences between conditions: feelings of refreshment, subjective sleep quality, bedtime, wake time, or total sleep time.

**Table 3:** Morning measure means, standard deviations, *F* values, 0.95 confidence intervals for condition mean differences, significance, and partial eta-squared effect sizes.

Variable		Rumination *M* (*SD*)	Compassionate reappraisal *M* (*SD*)	*F*	Mean difference 0.95 *CI*	*p*	*η* ^2^
*Sleep variables*	Bedtime	12.78 (1.25)	12.57 (1.72)	2.37	−0.06, 0.48	0.126	0.014
	Wake time	8.53 (1.24)	8.49 (1.21)	0.09	−0.19, 0.26	0.760	0.001
	Subjective sleep quality	3.48 (0.91)	3.48 (0.90)	0.01	−0.16, 0.17	0.945	<0.001
	Feelings of refreshment	2.66 (0.93)	2.78 (0.95)	1.81	−0.29, 0.06	0.180	0.011
	Total sleep time	7.46 (1.24)	7.55 (1.35)	0.52	−0.34, 0.16	0.473	0.003
	Trouble falling or staying asleep	0.37 (0.70)	0.22 (0.56)	7.12	0.04, 0.26	0.008	0.040
	Sleep onset latency	21.51 (22.13)	17.96 (17.61)	4.15	0.11, 6.99	0.043	0.024
	Sleep disturbances	27.95 (10.53)	25.57 (9.98)	11.70	1.01, 3.76	0.001	0.065
*State self-report scales*	Impact of event (IES)	4.51 (5.06)	2.70 (3.86)	40.13	1.24, 2.37	<0.001	0.192
	Rumination	12.43 (5.84)	10.58 (5.29)	38.38	1.26, 2.44	<0.001	0.185
	Accountability	38.65 (6.48)	42.75 (7.09)	102.02	−4.89, −3.29	<0.001	0.376
	Self-regulation	63.25 (13.18)	64.42 (13.54)	2.23	−2.72, 0.38	0.137	0.013
	Flourishing	33.94 (11.24)	34.23 (10.92)	0.50	−0.51, 1.09	0.479	0.003
	Eudaimonic	26.81 (8.79)	26.78 (8.49)	0.01	−0.60, 0.65	0.926	<0.001
	Hedonic	7.14 (3.05)	7.45 (2.93)	4.58	−0.61, −0.03	0.034	0.026

Sleep variables are based on the Sleep and Health Self Report Scale, with the exception of trouble falling or staying asleep (item 2 of the IES intrusion subscale). Possible score ranges are reported in the Measures section of the Method.

#### Psychological impact reported the next morning

Table 3 documents the subsequent morning impacts of rumination vs. compassionate reappraisal, largely consistent with hypotheses. Specifically, the morning after the offense rumination (vs. compassionate reappraisal) imagery and writing condition, participants reported greater intrusive impact of the offense event (IES) and levels of rumination, with lower levels of welcoming accountability to others. However, conditions did not differ for levels of self-regulation or total flourishing scores. To further investigate levels of flourishing between conditions, we conducted *post hoc* analyzes of flourishing subscales scores. This showed higher hedonic flourishing (feeling happy, interested in life, and satisfied)—but not eudaimonic flourishing—the morning after compassionate reappraisal.

## Discussion

The current study centered on trait and state assessments of variables relevant to the literatures on forgiveness and sleep, factors associated with biopsychosocial flourishing (Léger et al., 2001; Witvliet, 2020). In doing so, we used both an individual difference correlational design and a quasi-experimental design (comparing compassionate reappraisal versus rumination about an unresolved real-life offense) to replicate findings while also extending the literature.

### Individual differences related to forgiveness and sleep

Recent research showed that at the trait level, people who were more forgiving also slept better (Toussaint et al., 2019). Other research found that the forgiveness and sleep relationship was mediated by lower levels of rumination and negative affect (Stoia-Caraballo et al., 2008). The current study replicated this indirect effect. Specifically, the trait of being forgiving toward others indirectly predicted sleep quality in the past month through lower trait rumination and negative (depressed and anxious) affect, and through lower negative affect alone. This work further demonstrated that sleep difficulties over the past month were correlated with rumination, anxiety and depression, as well lower self-regulation and flourishing. By contrast, forgiveness was positively associated with self-regulation and flourishing, and negatively associated with depression and anxiety. Thus, the current work expands the literature on individual differences. In light of these individual differences, we also sought to test whether adopting one cognitive approach or another (ruminating or cognitively reappraising with compassion) would make a difference for states of forgiveness and sleep along with other psychosocial factors.

### Compassionate reappraisal, rumination, and sleep

We used a quasi-experimental design to replicate forgiveness-related findings while extending the literature to test sleep and other psychosocial variables. Specifically, this study replicated the empathy, forgiveness, social, and emotional effects of rumination about a real-life and unresolved offense in comparison to compassionate reappraisal (see Witvliet, 2020) while contributing new findings about biopsychosocial self-reported variables including sleep.

One reliable sleep effect was that sleep onset latency was delayed after ruminating rather than compassionately reappraising one’s real-life offender. This finding builds on previous research which showed that a less personal form of rumination (e.g., about a midterm exam) delayed sleep onset latency (Guastella and Moulds, 2007). In addition to delayed sleep onset, the rumination condition (vs. compassionate reappraisal) was associated with more sleep disturbances overall. Further, an item from the IES showed that after the rumination (vs. compassionate reappraisal) imagery and writing, participants perceived more trouble falling or staying asleep due to intrusive thoughts or images associated with the distressing event. Other indicators of sleep, however, did not show reliable differences (self-reported sleep timing, quality, or feelings of refreshment). Overall, sleep differences centered in falling asleep and sleep disturbances, with intrusive thoughts and images delaying and disturbing sleep. Thus, for people with offense rumination and these types of sleep difficulties, compassionate reappraisal imagery and writing prompts could offer a beneficial approach. This study also demonstrated that these cognitive approaches could be prompted, and self-reported responses could be assessed, through a virtual format. Results suggest that people who want to find a way to hold offenders accountable while also being more forgiving and accountable themselves may find that compassionate reappraisal provides a path forward.

### Compassionate reappraisal, rumination, and state forgiveness, emotions, and prosocial accountability

Further evidence across the linguistic measures, scales, and ratings showed that compassionate reappraisal (vs. rumination) significantly elevated forgiveness and empathy, positive emotion, and social responses. By contrast, ruminating about one’s offense activated more negative emotion, aroused/intense emotion, anger, sadness, and lower levels of perceived control. These findings are consistent with several studies finding that compassionate reappraisal (vs. rumination) improved forgiveness and emotional states (Witvliet et al., 2001, 2010, 2011, 2020; Baker et al., 2017).

The present study also showed that induced rumination on night one was associated with higher reports the next morning of intrusive thoughts of the offense and ruminations about it since the imagery and writing induction. These findings—in combination with the emotion effects—are broadly consistent with prior research which demonstrated that rumination is linked to state increases in anxiety (Olatunji et al., 2013) and depression (Aldao et al., 2010). However, rumination and compassionate reappraisal did not yield differences in self-reported self-regulation. Past research had shown that compared to a relaxing baseline period, rumination imagery impaired self-regulation as measured by heart rate variability, whereas compassionate reappraisal was equivalent to relaxation levels (Witvliet et al., 2010, 2011). Further, compassionate reappraisal promoted hedonic—but not eudaimonic—flourishing. That is, participants felt happier, more interested in life, and more satisfied the morning after, whereas their sense of societal connection, meaning, and purpose did not change.

Importantly, participants showed greater welcoming of accountability to fulfill their responsibilities to others—a prosocial response to give others what they are due (Peteet et al., 2022)—the morning after compassionate reappraisal compared to rumination. Interpersonal offenses have been interpreted as a failure of relational accountability (Witvliet, 2020), and this study offers the first evidence that a compassionate reappraisal (vs. ruminative) response to one’s real-life offender may elevate one’s own willingness to be accountable to others. It is possible that such willingness to be accountable to other people could diminish the likelihood of offending others—a possibility worth further study. Compassionate reappraisal is a response consistent with holding interpersonal offenders accountable for their offenses while desiring needed positive transformation—an approach that promotes forgiveness and positive side effects of improved emotions, sleep, and prosociality. Thus, this study amplifies evidence pointing to compassionate reappraisal as a response to offense rumination that is consistent with both justice and mercy, while promoting a suite of psychological, physiological, sleep, and social shifts important for mental and physical health and quality of life (Zlotnick et al., 2000; Léger et al., 2001; Witvliet, 2020).

### Study strengths and limitations

A strength of this study was its attention to both individual difference (trait) and state levels in assessing forgiveness, sleep, and related psychosocial variables. At the same time, longer interventions and longitudinal designs are needed to test whether people can implement compassionate reappraisal in ways that grow forgiving dispositions and enduring sleep and psychosocial benefits.

One strength of the online paradigm was that the imagery and writing components of the conditions could be standardized in format and timing. Further, collecting data in participants’ home environments elevated ecological validity by assessing night and morning effects in the residence setting in which the rumination, compassionate reappraisal, and sleep occurred. The remote methodology also made it possible to conduct a sleep study in the context of COVID-19. Yet, we acknowledge that in the pandemic, general anxiety levels have been elevated (Lee, 2020). We cannot know if this influenced our study findings, and we hope others build on the current study to replicate and extend it.

From an experimental perspective, the study was designed to compare two cognitive approaches to an unresolved real-life offense, and we offered interpretations of the results in light of the two conditions of rumination and compassionate reappraisal. Accordingly, we urge readers to be cautious in interpretations because the design does not compare these conditions to a control that was not focused on one’s unresolved offense. We did not include a baseline (e.g., no-imagery relaxation) night due to concern about the length of the study in light of the goal of avoiding weekend nights and mornings in this US college sample, as well as possible drop-off in completion rates, or implications of practice or fatigue effects. The reasons for sequencing rumination before compassionate reappraisal were that forgiveness interventions begin with reliving one’s offense before taking steps to build empathy for that offender (Worthington, 2020), prior research has shown that participants ruminate with more empathy for their offenders after compassionately reappraising them (Witvliet et al., 2015), and we believed it would be unethical to conclude the experiment with rumination in light of substantial evidence showing its adverse emotional, relational, and physiological effects (see summary in Witvliet, 2020). Therefore, to retain standardization, reduce the potential for confounds, and maintain ethical research practice, we utilized this specific sequence.

Finally, a strength of the trait study is that we used gold standard measures for forgiveness (TFS) and sleep (PSQI). A strength of the state study is that we used many of the same measures as in prior studies to allow for replication, and the same effects on anger, empathy, and forgiveness were found for both the scales and single-item state ratings. However, a limitation of the sleep measures is that they relied on self-report rather than physiological assessment. Having sleep lab measures for the intervention would be valuable for internal validity and objective verification, although this was not feasible for this study because of cost, access, and COVID-19 protocols. While sleep apps and watches and ambulatory monitoring devices present a measurement alternative, this approach was also not feasible for us in the context of the pandemic and timing to conduct this study of 180 participants within an academic year time frame. Relatedly, our sample was comprised of residential college students in the US, so we cannot generalize to other populations without substantial Western, educated, industrialized, rich and democratic (WEIRD) caveats (Henrich et al., 2010).

### Future research

In contrast to rumination, compassionate reappraisal prompted increased empathy, forgiveness, positive emotion, prosociality, and better sleep in the short term. In light of this, it would be important to assess the effects of a more sustained and developed intervention on dispositional forgiveness, empathy, rumination, and sleep. Accordingly, if individuals consistently practice compassionate reappraisal rather than ruminating about real-life offenders, the repetition of the responses could promote habit formation and development of trait behaviors (Lally et al., 2010) with the potential to decrease overall rumination and negative emotions, and in turn, improve overall sleep. If so, this is one pathway for improved health (Levenson et al., 2016). At minimum, we recommend compassionate reappraisal as an antidote to rumination and commend its inclusion in integrative approaches to sleep and biopsychosocial health.

The current study builds on a programmatic body of forgiveness research. For example, four psychophysiological studies showed transforming benefits of compassionate reappraisal imagery in contrast to rumination. Two of these studies showed that compassion also outperformed suppression of negative emotions about the offense (Witvliet et al., 2011, 2015), leading us to hypothesize that trying not to experience or express negative emotions about one’s offense by another person would not aid forgiveness, empathy, positive emotion, prosociality, or sleep. Two of these studies incorporated a benefit-focused reappraisal that promoted forgiveness as well as gratitude and positive emotion with effects on event related potentials in the brain (Baker et al., 2017) and cardiovascular regulation evident in improved heart rate variability (Witvliet et al., 2010). Further, a recent study used imagery plus writing paradigm similar to the current study, finding use of two consecutive compassion and benefit-focused reappraisals strengthened and sustained forgiveness and diminished negative emotion effects of rumination (Witvliet et al., 2020). Given the importance of rumination and negative affect in trait models, adding a third night-morning pair with the imagery and writing induction paradigm tested here—and counter-balancing the sequence of compassion and benefits—could yield more potent forgiveness with side effects for emotion, prosociality (e.g., accountability to others), and better sleep. We hope the current research and suggested future directions catalyze research on sleep in relation to compassion, forgiveness, and positive psychology.

## Data availability statement

The datasets presented in this study can be found in online repositories. The names of the repository/repositories and accession number(s) can be found at: https://osf.io/5b7h8 (Open Science Framework pre-registered hypotheses; associated project link https://osf.io/wr2f7/ contains protocol files and data sets).

## Ethics statement

The studies involving human participants were reviewed and approved by Hope College Human Subjects Review Board. The participants provided their written informed consent to participate in this study.

## Author contributions

AG and CW co-developed the research idea, protocol, HSRB, OSF preregistration, analysis plan, analyses, and manuscript. AG worked closely with all students involved in data collection. SB collected data, assisted with analyses, and contributed to all phases of the manuscript. All authors contributed to the article and approved the submitted version.

## Funding

This publication was made possible through the support of a grant from the Templeton Religion Trust (#TRT0171) awarded to CW. AG, SB, and Haley Balkema were awarded a faculty-student collaborative grant through the Jacob E. Nyenhuis program from the Donald W. Cordes Faculty Development Fund in order to provide funding for conducting data analyses on the project. Generous funding was also provided by the Van Wylen Library at Hope College to help defray publication costs.

## Conflict of interest

The authors declare that the research was conducted in the absence of any commercial or financial relationships that could be construed as a potential conflict of interest.

## Publisher’s note

All claims expressed in this article are solely those of the authors and do not necessarily represent those of their affiliated organizations, or those of the publisher, the editors and the reviewers. Any product that may be evaluated in this article, or claim that may be made by its manufacturer, is not guaranteed or endorsed by the publisher.

## Author disclaimer

The opinions expressed in this publication are those of the authors and do not necessarily reflect the views of the Templeton Religion Trust.

## Footnotes

^1^ https://osf.io/5b7h8

^3^ https://osf.io/wr2f7/

^4^ https://www.sona-systems.com

^5^ https://osf.io/5b7h8

^6^ https://osf.io/wr2f7

^8^ https://osf.io/5b7h8

^9^ https://osf.io/wr2f7
